# Evaluation of the Implementation of a 25-Year Outdoor School Ground Smoking Ban: A Qualitative Interview Study With Implications for Prevention Practise

**DOI:** 10.3389/fpubh.2021.628748

**Published:** 2021-04-01

**Authors:** Pia Kvillemo, Kristin Feltmann, Tobias H. Elgán, Johanna Gripenberg

**Affiliations:** Stockholm Prevents Alcohol and Drug Problems, Centre for Psychiatry Research, Department of Clinical Neuroscience, Karolinska Institutet, Stockholm Health Care Services, Region Stockholm, Stockholm, Sweden

**Keywords:** tobacco, outdoor school ground smoking ban, qualitative content analysis, policy, students, public health, Sweden

## Abstract

**Introduction:** Tobacco use, often initiated in younger ages, is a serious health challenge worldwide. In Sweden, smoking has been prohibited on school grounds since 1994. Municipal environmental and health inspectors control the compliance of the ban. Nevertheless, the enforcement and maintenance of the ban are inadequate. The aim of the current study was to identify facilitators, barriers, and the potential for improved implementation of a 25-year outdoor school ground smoking ban in upper secondary schools.

**Materials and Methods:** A process evaluation was conducted using semi-structured interviews with principals in upper secondary schools, local environmental and health inspectors, and local politicians (*n* = 30) in Stockholm County, with purposive sampling for informant recruitment. A qualitative content analysis of the transcribed interviews was performed.

**Results:** Three main categories and 10 subcategories were generated from the interviews, revealing facilitators, barriers, and the potential for improvement of the implementation of the ban. A prominent facilitator of the ban was informed and engaged principals and inspectors. Prominent barriers were conflicting goals governing the schools, which reduce staffs' motivation to maintain the ban, unclear school ground boundaries, and lack of resources. Potential for improvement was found in a new tobacco act with an extended ban on smoking at school entrances, extended support for schools and staff to strictly enforce the ban, and a continued denormalisation of smoking in society.

**Conclusion:** To achieve effective implementation of outdoor school ground smoking bans in upper secondary schools, authorities need to address conflicts between different goals governing the schools and give necessary support to the staff to strictly enforce the ban. Policies on smoke-free working hours in the municipalities along with tobacco restrictive policies in the surrounding society may increase the possibility to maintain the smoking ban in upper secondary school grounds.

## Introduction

Tobacco use is a serious public health challenge in the world. According to the World Health Organisation, one in ten deaths is caused by tobacco use (1). Smoking is associated with several diseases, such as chronic obstructive pulmonary disease, cardiovascular disease, lung cancer, and other cancers (2–4). Therefore, most countries, including Sweden, have adopted restrictive policies on tobacco sales and tobacco use (5–8). Smoking at an early age when the brain is under development is particularly harmful and associated with being an adult smoker (9–13). European data from 2015 on students aged 15–16 years revealed that 21% were smokers (range between countries 6 and 37%) (14). In line with these data, a Swedish, school survey from 2018 showed that 23% of the students aged around 17 were smokers (in upper secondary school) (15), calling for action. Since young people spend a lot of mandatory time in school, the implementation of smoking bans in school settings has been regarded as a promising policy intervention to prevent smoking among children and young adults. Thus, many countries have in the last decades implemented smoking bans in school settings (5, 16–20). Studies on the direct effect of such bans have, however, shown inconclusive results with regard to students' smoking behaviour (20–25). Thus, there is a need for more research on how the bans function in relation to the level of enforcement, national setting, and possible indirect effects (17, 18).

In the growing field of implementation research, some general knowledge on effective implementation strategies has emerged. In a comprehensive synthesis of the literature, Fixsen et al. (26) argue that implementation activities are influenced by components connected to the implementation process (e.g., training and coaching of staff), the organisation in which an intervention is implemented (e.g., prioritisation, attitudes and norms among staff), and external circumstances (e.g., societal norms, politics, and economy). Moreover, they claim that an implementation process, in general, takes between 2 and 4 years and contains the following six stages: (1) exploration/adoption, (2) installation, (3) initial implementation, (4) full implementation, (5) innovation, and (6) sustainability. Similarly, Scaccia et al. (27) emphasise the importance of innovation-specific capacities, motivation to implement an innovation in an organisation, and general capacities of the organisation in which it is implemented. Referring to Aarons et al. (28), Scaccia, et al. (27) also highlight the need to ensure quality implementation throughout the entirety of the innovation's lifespan.

Outdoor school ground smoking bans are internationally less common than corresponding indoor smoking bans, especially those regulated by law at a national level (16, 18, 24). Thus, less is known about the facilitators and barriers influencing the implementation of these bans. The few studies that we found on facilitators and barriers affecting the implementation of outdoor school ground smoking bans specifically were from the Netherlands (16, 29) and Canada (30, 31). While studying the sustainability of an outdoor school ground smoking ban in the Netherlands, Rozema et al. (29) found that perceived barriers and facilitators for sustainability could be sorted into the following three categories: smoking ban implementation factors, school factors, and community environment factors. The authors concluded that the involvement of all staff is important for sustainability, as they function as role models, have an interrelationship with students, and share responsibility for enforcement. In a process evaluation of the implementation of an outdoor school ground smoking ban in 24 Dutch secondary schools, Rozema et al. (16) consolidated the findings on facilitators and barriers (1–3) outlined above, suggesting that directors should deal with violators by strictly enforcing the ban, initiating a dialogue with them, and/or using counselling as methods to help offenders stop smoking. In a Canadian study on the enforcement of a ban on smoking on school properties, Ashley et al. (30) found that schools and tobacco enforcement officers experienced a lack of resources and an increased workload in schools, as a result of the enforcement task given to them. The authors also cited the need for increased education on tobacco for students as well as complementary measures such as price increases and enforcement of the ban on sales to minors. Finally, in another Canadian study of a smoking ban in schools, Pickett et al. (31) suggested that attention should be given to informing teachers and gaining their support when implementing a ban, as well as having strategies for dealing with potential safety risks to students who leave the school property to smoke.

In Sweden, a national ban on smoking on school grounds was established by law in 1994 (32). The responsibility for ensuring compliance with the ban is divided between authorities at the national, regional, and local levels (33). The Public Health Agency has central supervisory responsibility at the national level, while the County Administrative Boards have a regional responsibility, and the municipality authorities have a local supervisory responsibility. The County Administrative Board shall follow the municipalities' activities and assist them with information and advice, and promote cooperation between relevant organisations and actors. The municipality's environmental and health inspectors conduct supervision of compliance with the tobacco legislation at the municipal's schools. The inspectors can instruct the school to ensure that smoking at the school ground ceases and request an action program. The inspectors can also suggest changes in the physical environment, e.g., removal of ashtrays, and initiate injunctions or prohibitions, sometimes combined with an economic penalty (33, 34). Injunctions and prohibitions must be approved by local politicians, who have responsibility for smoking policy in their municipality, which is often carried out by a department handling social and health-related issues. The municipality is responsible for the supervision of several environments in the community and can charge fees for supervision connected to that. However, they cannot charge a fee for the supervision regarding the outdoor school ground smoking ban. The primary responsibility for maintaining a smoke-free school ground lies with the school's principal (34). Tobacco prevention activities, like information to staff, students and parents at schools, can be assisted by actors in the local community, including staff employed by the municipality, e.g., prevention coordinators. In 2016 (34), prevention coordinators were found in almost all of the 26 municipalities in Stockholm County, as well as in the city districts of Stockholm.

Despite efforts to ensure that the smoking ban on school grounds, launched in 1994, is properly implemented, the enforcement of the ban was still found inadequate in 2016–2018 (34–36). Surveys among school staff in Stockholm in 2016 and 2017 suggested that smoking occurs on several of the county's school grounds, not least in upper secondary schools, suggesting the need for effective actions to improve the implementation of the ban (34, 37).

Previous studies on facilitators and barriers influencing the implementation of smoking bans in school settings, including those focusing on outdoor school ground smoking bans, have investigated the implementation process primarily from a school perspective. One exception is a recent study by Hoffmann et al. (18), assessing barriers to the implementation of school tobacco policies (indoor and outdoor) in seven European cities by interviewing local stakeholders outside the schools. In the current study, we build on previous research by combining three perspectives on the implementation of an outdoor school ground smoking ban, namely the perspective of key stake holders, i.e., secondary school principals, local politicians, and municipality inspector officials. The aim of the current study was to identify facilitators, barriers, and the potential for improved implementation of a 25-year outdoor school ground smoking ban in upper secondary schools in Stockholm County. The study will add to a multi-perspective knowledge base of the implementation processes related to outdoor school ground smoking bans and highlight necessary measures to improve the implementation and maintenance of such bans.

## Materials and Methods

A process evaluation study was conducted using qualitative data from semi-structured interviews with local stakeholders in Stockholm County.

### Participants and Procedures

Purposive sampling was used to include relevant informants for the interviews, based on the organisation of tobacco restriction control in Sweden. Three vocational groups were selected: principals in upper secondary schools (hereafter named principals), local environmental and health inspectors (hereafter named inspectors) and local politicians, elected and currently active (hereafter named politicians). The County Administrative Board in Stockholm provided the research team with a list of contact details to 30 local persons from each vocational group, i.e., 90 names from different municipalities and schools, including assisting principals. Persons were chosen from a variety of schools and municipalities to allow for varying sociodemographic profiles and efforts to implement the outdoor school ground smoking ban. To achieve reasonable saturation (38), the aim was to interview 10 persons from each category (*n* = 30). At the beginning of 2019, the research team chose the names of the first 10 principals, inspectors, and politicians on the list, asking them *via* e-mail to participate in the study. They were informed that participation was voluntary; that data would only be reported in aggregate form; and that audio and text files were to be stored safely (i.e., at encrypted servers) in coded form to preserve confidentiality. Informed consent was obtained by asking the receiver to reply, and if giving a positive answer, agreeing to participate in the study. If participants refrained from participation, additional persons were contacted, i.e., the next person on the list, until the intended number of participants was reached. About one-third to one-half of those contacted refrained from participation, mainly due to time constraints.

At inclusion of the participants, a code key was established with an individual code for every informant, consisting of a number between 1 and 10 connected to the current category, e.g., Principal 3, Inspector 7. The Regional Ethical Review Board in Stockholm was contacted and informed about the study but regarded it as unnecessary to review for approval (dnr. 2019-00719).

### Semi-Structured Interviews

Semi-structured interview guides for each category of informants were elaborated by the research team in collaboration with officials at the County Administrative Board. The interview guides included 10–12 questions, along with supplementary questions. The interviews started by asking the informants about how long they had held their current position, which was on average 3 years for inspectors, 6.5 years for principals, and 1.5 years for politicians. The main interview questions reflected issues concerning facilitators, barriers, and the potential for improvement of the implementation processes. Example questions were: *Initially, do you want to say something about the municipality's work with smoke-free schoolyards? In what way does the school inform students about the smoking ban? Are smoking and the health risks something that is integrated into teaching? What else would be required to remove smoking on the school grounds?* Three of the authors (KF, THE, PK) conducted the interviews by phone. The interviews, on average 20 min long, were recorded and transcribed verbatim into 272 pages of text.

### Content Analysis

Qualitative content analysis with a team-based approach was used to analyse the interview data (39–41) and the software NVivo 12 was utilised for structuring the data. The four researchers in the team have extensive knowledge in social sciences, public health, drug prevention, and implementation theories, which facilitated the analysis. Initially, one researcher (PK), who has a PhD in medical science, a master's degree in political science, and extensive experience in qualitative analysis, started the analysis process by repeatedly reading 8 of the 30 interviews. The analysis was to some extent deductive since the interview questions initially directed the analysis (40). During the reading, meaningful units were identified and grouped into categories (Supplementary Table 1). A preliminary coding scheme with the key concepts of facilitators, barriers and potential areas for improvement, along with main categories, inspired by Rozema et al. (29), and sub-categories with definitions (codebook) (41) were developed and presented for the other three researchers in the team (KF, TE, JG). The team discussed the definitions of certain codes and agreed to a slightly refined codebook, but with the same number of codes. After the discussion, PK, continued the coding of the remaining interviews, while defining the various codes more clearly, excluding one of them because of irrelevance, and dividing one sub-category of codes into two. To assess the reliability of the coding, an independent recoding of three of the interviews (one from each occupational group) was carried out by a second coder from the research team (KF) (42). A high degree of agreement between coders was obtained with a few disagreements resolved through discussion.

## Results

### The Informants

The final group of informants represented 17 of the 26 municipalities in Stockholm County, including municipalities with various sizes and socioeconomic characteristics (43, 44) (Supplementary Table 2). The capital of Sweden (Municipality “A”) is by far the largest municipality with the most schools within its borders, both public and independent. With the exclusion of this municipality, the mean size of all municipalities in the county is 52,056 (Standard Deviation (SD): 28,131.51) inhabitants and the mean number of upper secondary schools 4.2 (SD: 3.84) of which 1.64 (SD: 1.38) are public schools and 2.68 (SD: 3.03) are independent schools. The corresponding numbers for the municipalities included in the study, excluding the municipality of Stockholm, are 62 413 (SD: 28,807.92) inhabitants, 5.56 (SD: 4.15) upper secondary schools, 2.12 (SD: 1.49) public schools, and 3.24 (SD: 3.56) independent schools. Thus, the municipalities included in the study are on average somewhat larger than those not included. In terms of education, the proportion of inhabitants aged 25–64 years with at least 3 years of university education is 31% in the entire county, and in the municipalities included in the study 32%. Eight principals, two representatives of principals, i.e., one administrative manager and one official, appointed by the principal, 10 inspectors, and 10 politicians, constituted the final group of informants.

### Analytical Categories

The key concepts, facilitators, barriers, and potential factors were categorised into three main categories, corresponding to those previously outlined by Rozema et al. (29), i.e., smoking-ban implementation factors, school factors, and community environment factors. Smoking-ban implementation factors are factors related to (1) the actual implementation of the ban, including how it is expressed in the Tobacco Act, (2) enforcement actors at different organisational levels, and (3) the enforcements' actor's performance and ability to implement the ban. School factors are factors connected to the schools *per se*, regardless of the smoking ban. Community environment factors are circumstances which surround the actual implementation organisation, influencing the implementation of the ban without being directly connected to the implementation process. Along with the main categories, 10 sub-categories were defined, as outlined in Table 1.

**Table 1:** Main categories and sub-categories.

**Main categories**	**Sub-categories**
Smoking-ban implementation factors	Regulation of outdoor school ground smoking ban by The Tobacco Act
	Enforcement actors
	Municipality-based control of compliance with outdoor school ground smoking ban
	School leadership
	School-based implementation and enforcement
School factors	School culture
	School ground
Community environment factors	Other actors or activities in the society
	Social environment
	Laws and regulations apart from the smoking ban on school grounds

Additionally, 29 codes were generated from the material (Supplementary Table 3). Facilitators, barriers and potential for improvement were found in all the sub-categories, except for “Laws and regulations apart from the smoking ban on school grounds,” were no facilitators were revealed. In the following, the results are presented under headings corresponding to the main categories and sub-categories.

### Smoking-Ban Implementation Factors

#### Regulation of Outdoor School Ground Smoking Ban by the Tobacco Act

Although a smoke-free school ground is regulated by law, only one informant emphasised that a ban facilitates the maintenance of non-smoking in upper secondary school grounds. Several other informants found it difficult to implement the ban and the view that the law is meaningless because of a lack of tools to maintain compliance with it was also expressed.

*It doesn't help us, because nothing happens, and our students know that if they smoke in the school ground, “it is certainly forbidden by law, but I don't suffer the consequences.” So, that it is forbidden by law, will not be a tool for us. Possibly signal policy, but at least our young people are not receptive to that type of signal policy, so I don't think the law helps me at all*. (Principal 3)

At the time of the interview (January–March 2019), several informants looked forward to a revised version of the Swedish Tobacco Act that was due to be implemented in July 2019. The new act encompasses a smoking ban in certain public spaces, including the entrances to school buildings, which several informants said will make it easier to enforce the ban, especially at schools without a well-defined school ground.

#### Enforcement Actors

A facilitating factor related to enforcement actors concerned support given to the municipalities by the County Administrative Board. Meetings organised by the Board are perceived as rich opportunities to learn about the interpretation of the Tobacco Act and how to write injunctions and prohibition documents regarding deficiencies in compliance with the school ground smoking ban. The inspectors further expressed appreciation for the opportunity to meet colleagues and exchange experiences at the meetings.

*I think we've got a lot of information. We've had a lot of fun. We have received manuals, manuals on policy, handbooks on action plans. We have checklists. We had a network meeting last autumn at the County Administrative Board*. (Inspector 10)

Most of the inspectors expressed engagement and motivation for their work and suggested that managers were also engaged in the tobacco prevention work. Although the work was perceived as meaningful, two of the inspectors doubted whether they would succeed in abolishing smoking at the school grounds.

*I think it feels meaningful […]. But there are cases when it may not really work. Thus, upper secondary school students get so much input from so many other “realities.” Both from social media and television and the mass media*. (Inspector 8)

Enforcement actors expressed that barriers mostly seemed to concern local politicians, expressed by a local inspector in the following way:

*The politicians in our local committee, they are interested in issues on building law and beach protection. But in tobacco, I can say, they are not interested at all!* (Inspectors 8)

Some politicians described smoke-free school grounds not to be a prioritised issue in the municipality, while a majority said that smoking among young people is important to address, although smoke-free school grounds cannot be prioritised over other required municipality tasks. In line with this, only a few municipalities have implemented smoke-free working hours for municipality employed staff. One politician saw an opportunity to educate local politicians about tobacco and related problems in order to facilitate well-informed decisions on the issue.

#### Municipality-Based Control of Compliance With Outdoor School Ground Smoking Ban

The frequency of supervisory visits at schools varies between municipalities, with some schools being visited every year or every second year and others less frequent. The absence of a specific fee for supervision is considered to contribute to sparse visits. However, half of the inspectors considered the existing frequency of supervision to be sufficient. Additionally, two inspectors considered injunctions to be a useful tool for supervision.

*It gives more weight […] than if it becomes a report that may end up in someone's junk mail or the like. […] We have that opportunity and I think we should use it*. (Inspectors 8)

The view on injunctions among principals was overall neutral or negative. Several inspectors expressed reasons why these instruments are not used more often.

*We find it very difficult to submit injunctions about smoking to a school. This is just about a school ground, what is a school ground? Then you can always ask for routines, but they often have them*. (Inspectors 6)

The possibility of adding a charge to injunctions and prohibitions was regarded as problematic by some politicians and inspectors, partly because it would seem strange to demand payment from an actor financed by public funds.

*No, I don't believe it. […] Even if you were to charge a private school, it's tax money that the school is running with. It doesn't feel reasonable*. (Politician 2)

Some inspectors indicated that supervision of schools through more frequent visits would improve the schools if there were more resources assigned for the visits. However, there were also inspectors claiming that that with more resources they would prefer to focus on preventive supervision than on supervisory control visits. Smoke-free working time in all public workplaces in the community, including schools, was also suggested as more effective than making more supervisory visits to schools.

#### School Leadership

All principals seemed well-informed about the outdoor school ground smoking ban and could also express reasons why the ban exists, such as smoking being a health hazard and non-smoking students and staff should not be exposed to or inhale tobacco smoke. A majority of the principals argued that a smoke-free school ground is prioritised even if it is not the highest priority on the agenda.

*Yes, it's quite a priority. Obviously, there are degrees in everything. It may not be the primary mission, but it's a priority. It's something we work with daily*. (Principal 1)

Both principals and inspectors stated the schools generally comply with observations on the shortcomings and requirements of inspectors. Activities linked to tobacco prevention in the schools include teachers and other staff who see students smoking on the school ground and tell them to stop. Although most principals seemed motivated to carry out the implementation of a smoke-free school ground, there was also a critical stance on the school's responsibility for maintaining the tobacco law's smoking ban.

*To put all the responsibility on the school by having a law that says it's forbidden to smoke on the school ground, without adding any resources at all. [...] It doesn't work! I have been working at a lot of different upper secondary schools and it has never worked. […] Young people do dangerous things and it doesn't help with a ban. Period*. (Principal 3)

When trying to stop students from smoking on the school ground, staff sometimes experience problems, such as rude responses from students and a lack of time to stop and argue. Two principals expressed the problem as follows:

*That's easier said than done [...] The one who stops may not know who the student is that stands and smokes. It's not always certain that he or she will agree to leave the area. Sometimes they choose to remain, or even perceive a kind of provocation, acting rude*. (Principal 1)

*We have no chance whatsoever of having systematic control, or hiring someone who walks around as a smoke guard at the school. […] We don't have the capacity within the school budget, to stick with employees who have this as an assignment*. (Principal 2)

Two of the principals thought that they were required to work harder to raise the staff's sense of responsibility to act when observing smoking students. Simultaneously, they highlighted the insufficient sanctions they can employ if smoking students are discovered. The principal and staff can tell students to stop and inform the parents that their child is smoking if the student is under 18 years of age, but otherwise, the student has to approve the contact with parents. The school can send one or more warnings to parents or students themselves if they are of legal age, but then several principals feel that it is impossible to do more. However, several principals said that suspension due to smoking is not possible within the framework of the Education Act and the curriculum.

*What do we do if we catch a student who smokes in the school ground? […] At the individual level, we cannot suspend students because they smoked on the school ground*. (Principal 4)

Further, some principals believed that prohibition is not the right way to achieve smoking cessation on the school grounds and conflicts between staff and students due to the students' refusal to stop may inhibit learning and completion of students' education.

*My assignment is that the students should be in school and learn things, and then it feels very unreasonably to expel a student. […] It will be like a kind of punishment that may somehow affect the student's studies and study results*. (Principal 6)

Regardless of the possibility of suspension, some principals believed that conflicts between staff and students over smoking habits are not worth the problems that they create. One principal, however, held a strict position regarding the smoking ban, stating that it is clearly a school's task to ensure that no smoking occurs on the school ground and that schools should take action and stand behind the law. Claiming the potential for schools to effectively address a violation of the ban, the principal expressed the following:

*The school must own this issue and regard it as important. We, the school, stand behind this law, and that's a thing that one should work for*. (Principal 9)

This principal claimed that there was no, or almost no, smoking occurring on the school ground currently, in contrast to the remaining principals who clearly stated that smoking occurs at their schools.

#### School-Based Implementation and Enforcement

Principals indicated that all schools have a policy on tobacco and smoking. Moreover, smoking prohibition is communicated *via* signs on school grounds. The tobacco policies include information, e.g., that smoking is prohibited in the school's area, the harmful effects of smoking, what measures the school should take if students violate the smoking ban and other measures to be implemented against smoking. The prevention efforts at schools include, e.g., integrating teaching on tobacco and related consequences into the schedule, smoking cessation support, and various campaigns and programs. Information about policy and any action plan or rules of procedure regarding smoking is preferably given when the students are introduced to the school, often accompanied by information to their parents. Education about smoking and related issues is further often integrated into the curriculum. One of the principals said that parental contact, in case students violate the smoking ban, can have the intended effect on students' smoking. However, since the parents often already know that their child smokes, the contact often does not improve the situation but is rather perceived as a time-consuming process.

*But just to take this to the next step and try to register the incident in some way. And then send home a warning to parents of minor students, or to the student, if they are of age, I think is an extremely difficult procedure*. (Principal 1)

Other principals expressed a pragmatic view of the problem, saying that it is good that staff continue to tell students that smoking on the school ground is forbidden even if no additional measures are taken when students are caught smoking. Conversely, one principal, the same principal who had obtained a smoke-free school ground, said that it is extremely important to maintain a restrictive line against smoking and expressed potential for intensifying implementation efforts as follows:

*You have to inform with clarity in writing! Both orally and written […]. The students know that it will be a consequence. They know that many adults care. They know that adults are caring. They know that if they are under the age of 18, we call home*. (Principal 9)

### School Factors

#### School Culture

Several principals expressed an awareness that staff are role models for students in smoking prevention. Some schools had made efforts to support the staff with smoking cessation and promote a smoke-free school. Despite the awareness of staffs' importance as role models for the students, some informants had observed staff smoking on the school ground, e.g., school canteen staff and teachers. Additionally, students can often see other adults smoking near the school ground, or even in the school ground, as many schools share the area closest to the school with other organisations with no smoking ban. Moreover, students can see smoking peers, either on the school ground, at entrances, or next to the school ground, inhibiting a supportive school culture. The social aspect of smoking and the influence of peer behaviour on students' was also highlighted.

*I would say that it's very much a social phenomenon. It's not often I see a student standing and smoking alone, rather going out together*. (Principal 2)

In order to prevent students from smoking, some schools supply other social activities, such as sports equipment and games.

#### School Ground

The presence of a school ground is central for the possibility of enforcing the school ground smoking ban. Several schools have no school ground at all and those that do often have an unclearly defined ground, which was expressed as problematic.

*There has always been a dilemma. What is a school ground and what is not a school ground? It's very difficult in some places*. (Inspector 8)

Due to supervision from the inspectors, several schools have removed smoking shelters and ashtrays from the school ground to prevent smoking. Nevertheless, the issue of having ashtrays or not is disputed. Some informants argued that it is better for smoking students to put butts in ashtrays than on the ground, to prevent an unpleasant environment. Some inspectors had, despite guidance to avoid objects or circumstances that invite smoking, observed the presence of smoke shelters or hidden places in some school grounds. A conflict between a school's sometimes more permissive attitude to smoking and the inspectors' more restrictive perspective was expressed by one of the principals as follows:

*We talked before about having ashtrays outside the school's area, so that we can really clearly mark that “if you should smoke you will smoke there.” But we didn't get permission for that from the municipality environment and health unit. And due to that, a lot of butts end up on the ground, unfortunately*. (Principal 5)

One of the principals suggested that making the school ground outside smoke shelters more pleasant and beautiful would encourage students not to smoke but stay in the nice areas of the school ground.

### Community Environment Factors

#### Other Actors or Activities in the Society

In addition to the school and the municipality's supervisory organisation, the informants see the surrounding community as an important factor in achieving smoke-free school grounds. This includes the municipality's other activities, e.g., leisure activities, crime prevention, and drug prevention. The collaboration with prevention coordinators and the role of social services were cited as a resource in tobacco prevention at the schools.

Working within the school on different levels and with outside support to prevent smoking was highlighted by one inspector, who gave observations of what an ideal prevention effort might look like.

*When you go to such good schools with colleagues that work on it on many levels, they may have had tobacco-free school time. And there has been contact with parents, counsellors and it has been possible to offer cessation weaning talks. And so, you have people who can help and support*. (Inspector 1)

Several informants emphasised that the school needs additional external support from decision-makers and authorities.

*The school obviously needs guidance, an idea bank, something to stand on. […] that they feel that they have a support. In part from the decision-makers, but above all, from government agencies that can produce information material*. (Politician 2)

#### Social Environment

Despite an increasingly more tobacco-restrictive norm in the society, several informants referred to social norms at both group and societal levels as an explanation for adolescents' and adults' smoking. To change the attitude of young people to a more tobacco-restrictive orientation, the idea of a collaboration between school and parents to convey preferable values was advanced. The influence of peers as an important factor for whether students start smoking was also highlighted.

*Students starting in one year, and returning in the second year, suddenly have started smoking. And it's very much about group affiliation and identity and who you want to keep up with*. (Principal 4)

Several of the informants said that changed norms throughout society are necessary to make young people abstain from smoking.

#### Laws and Regulations Apart From the Smoking Ban on School Grounds

Several principals indicated there are insufficient sanction opportunities connected to the school ground smoking ban, and the combination of the requirements of the Tobacco Act and the Education Act along with the school curriculum is also a problem. One principal expressing frustration suggested a change in the Education Act or the Tobacco Act.

*I would like it to be included in the School Act or the Tobacco Act, anywhere. I would like some clearer powers to actually take any kind of disciplinary action. Because I think it's terrible to go on forever [telling smoking students to stop]*. (Principal 1)

Several of the informants mentioned that sharper tobacco legislation in general, which includes areas outside the school, is needed to eliminate smoking in society and thereby in schools. One reasoned about a total ban in society as follows:

*So, to get rid of smoking, because that is what you really should work with, you simply have to criminalise tobacco possession and use. And the state should simply cease with this double standard that we have the smoking as a tax source for our welfare system, while also saying that we should stop smoking*. (Principal 1)

## Discussion

The aim of the current study was to identify facilitators, barriers, and the potential for improved implementation of a 25-year outdoor school ground smoking ban in upper secondary schools. The most salient finding in our study is the conflicting goals governing the schools, which seem to lower staffs' motivation to maintain the ban. Moreover, in upper secondary schools in Stockholm County, the lack of school grounds or unclear school ground boundaries impede the implementation of the ban.

Several factors promote the implementation of the outdoor upper secondary school ground smoking ban in Stockholm County. Consistent with previous research, facilitators are good support from the County administrative board; collaboration between local community actors; tobacco prevention strategies in schools; education and other prevention activities for students; well-informed principals engaged in the prevention of smoking on the school ground (26, 27), although to a somewhat varying degree; and generally motivated inspectors with access to sharp sanction tools (26, 27), although they are not always used. However, smoking on upper secondary school grounds in Stockholm County is a persistent problem, and the work to achieve smoke-free school-grounds is hampered by several barriers.

Firstly, there is a lack of fit between existing values and policies governing the school (45), vs. the outdoor school ground smoking ban in the Tobacco Act (32), which is an implementation barrier highlighted in previous research (26, 27, 46, 47), but not specifically in studies on the implementation of outdoor school ground smoking bans (16, 22, 29–31). Staffs' positive attitude to the implementation is hindered by an obvious conflict between the goal of strict enforcement of the ban, connected to sometimes troublesome discussions with students and time-consuming routines to report violations of the ban, and the schools' overarching objective of students' learning and completion of their exams (45, 48). Hesitation to start conflicts with students and the lack of effective sanctions to use for violators (45), especially students of legal age, reduces the motivation to pay attention to and report smoking students to the school administration. Lack of motivation among school staff, although not explicitly linked to conflicting goals governing the schools, has previously been highlighted in research on the implementation of outdoor school ground smoking bans in Canada and the Netherlands (16, 29, 31, 47), and implementation research in general (26, 27). Teachers' goal of avoiding conflicts with students is generally positive, since good relations between students and staff can promote the implementation of outdoor school ground smoking bans (21, 22, 29, 47). However, this motivation seemed to cause a less strict implementation of the ban in the current study. Moreover, if more effective sanctions were introduced, such as suspension from school, it may adversely affect the students' studies, thereby lowering the staffs' and principals' motivation to use it. Nevertheless, it is important for stakeholders, including schools' principals and staff, to discuss the conflicts and find reasonable solutions to solve them.

Secondly, a conflict may exist between the goals of reducing smoking among young people in general (5), and achieving smoke-free upper secondary school grounds. If smoking students are forced to leave the school ground, they escape the influence of non-smoking peers and staff who express a non-smoking attitude and can also encounter people and situations that can be harmful (31). Therefore, forcing smokers to leave the school ground may be a less desirable solution to the problem of smoking students on the school ground.

Thirdly, despite seemingly clear and comprehensible legislation, some complexity (26, 27) arises in the Swedish outdoor school ground smoking ban due to unclear boundaries of upper secondary school grounds and even a lack of such school grounds, which is a barrier that, to our knowledge, has not been presented in previous studies (16, 22, 29–31). At schools where this issue exists, the principal and staff cannot point out the school ground boundaries for students, and inspectors visiting these schools cannot carry out proper control of compliance with the ban, which, of course, inhibits the enforcement of the ban. Some informants in the current study mentioned the possibility of combating school ground smoking in a more general manner, through the introduction of smoke-free working hours at public workplaces in all municipalities (21, 29, 49). This strategy has been successfully tried in some Irish, German and Finnish schools (50).

Fourthly, in line with previous implementation research in general (26, 27) and studies on the implementation of smoking bans in particular (16, 30), the lack of resources is perceived as an implementation barrier among principals and inspectors. Several informants expressed that the implementation of the outdoor school ground smoking ban is less of a priority compared to many other issues that must be handled within existing resources and working hours.

Despite several barriers, there seems to be the potential for improvement of the implementation of the Swedish outdoor upper secondary school ground smoking ban. A new tobacco act, implemented in Sweden in 2019, can likely prevent students from smoking at school entrances, regardless of whether or not there is a school ground, which decreases the importance of clear school ground boundaries. This policy change may also support the denormalisation of smoking, thereby facilitating the prevention of smoking among students (21, 29, 49). Sustaining a clearly restrictive non-smoking norm, supported by the new tobacco act, seems important and also possible, preferably in combination with the continuation of the existing education on smoking in the curriculum (24, 29, 51, 52). However, previous studies have also indicated that a high level of conflict between staff and students regarding the enforcement of smoking bans in school settings may have a reversal effect for some individuals (22, 52), especially for alienated students with a high risk of substance use and school drop-out (22). Rozema et al. (16) suggested that one way to lower the level of conflicts with smoking students is by training staff on effective ways of dealing with violators of the ban. Such training could be part of the solution in Swedish upper secondary schools. Also, other vocational categories could be employed or provided from the municipality to support the staff in the maintenance of the ban, e.g., prevention coordinators.

Collaboration between the municipality's committees and administrations and extra resources for tobacco prevention activities (26, 27) may also facilitate the work of achieving non-smoking school grounds. Education of politicians could facilitate an awareness of the importance of obtaining smoke-free school grounds, thereby facilitating a higher priority of current prevention efforts in the municipalities (26). Finally, the surrounding community and actions at the societal level can facilitate smoking prevention among students in several ways. Extending the smoking ban to areas near the schools might facilitate implementation, as highlighted in previous research (21). Tobacco restrictive norms among parents, and the prohibition of smoking at restaurants and public places can further contribute to the denormalisation of smoking and the reduction of teenage smoking (49, 53, 54). Moreover, compliance with the age limit for tobacco sales can make it more difficult for people below 18 years of age to obtain cigarettes, thereby preventing smoking both in schools and elsewhere (23, 55). A more drastic measure is a total ban on tobacco in the society, which would certainly lower the prevalence of smoking among students if the black market was effectively counteracted (21, 23).

### Strengths and Limitations

The current study has several strengths. Firstly, the inclusion of three vocational groups with the power to influence the implementation of the outdoor upper secondary school ground smoking ban provided rich material that deepens our understanding of the implementation, highlighting conflicting interests and different views connected to different professional roles. Secondly, the inclusion of informants from larger and smaller municipalities with different average education levels among inhabitants, which has been previously shown to influence smoking habits (56), reduced the risk of receiving a limited view of the implementation due to the exclusion of factors related to inhabitants' smoking habits, e.g., socioeconomic background (57), and municipalities' economic resources. Thirdly, the selection of municipalities and upper secondary schools with varying motivations to implement the smoking ban, as evaluated by the County council, may have influenced stakeholders to have a nuanced view of the implementation. In addition, the team-based analytical process, involving all the authors and applying independent coding, should strengthen the credibility of the results (58). However, there are also some limitations. Because less engaged persons in the implementation of the ban might be more likely to abstain from participation in the current study than more engaged persons, there is a risk of selection bias due to personal interest, resulting in a more positive view of the implementation of the ban than is actually the case. Future studies could include longitudinal statistics on progress, e.g., number of non-smoking policies in schools, and number of smoking students. Finally, the current vocational categories of informants may have given a too limited view on the problem with implementation of the outdoor school ground smoking ban. Future studies could include additional vocational categories, e.g., teachers and also students, to give an even more elaborated view on the implementation, e.g., concrete strategies to support the teachers and ways to approach smoking students.

### Conclusion

To achieve effective implementation of outdoor school ground smoking bans in upper secondary schools, authorities need to address conflicts between different goals governing the schools and give necessary support to the staff to strictly enforce the ban. Policies on smoke-free working hours in the municipalities along with tobacco restrictive (58) policies in the surrounding society may increase the possibility of maintaining the smoking ban in upper secondary school grounds. Future studies could include additional informants connected to the schools, such as teachers and students, and longitudinal data on smoking in upper secondary school grounds where smoking bans are implemented.

## Data Availability Statement

Collected data will be available from the Centre for Psychiatry Research, a collaboration between the Karolinska Institute and Region Stockholm, but restrictions apply to their availability, as they were used under ethical permission for the current study, and so are not publicly available. However, data are available from the authors upon reasonable request and with permission from the Centre for Psychiatry Research.

## Ethics Statement

Ethical review and approval was not required for the study on human participants in accordance with the local legislation and institutional requirements. The patients/participants provided their written informed consent to participate in this study.

## Author Contributions

PK: conceptualization, methodology, investigation, data curation, formal analysis, project administration, writing—original draft, and writing—review and editing. KF: conceptualization, methodology, investigation, data curation, formal analysis, validation, and writing—review and editing. TE: conceptualization, methodology, investigation, formal analysis, and writing—review and editing. JG: conceptualization, methodology, formal analysis, supervision, funding acquisition, and writing—review and editing.

## Conflict of Interest

The authors declare that the research was conducted in the absence of any commercial or financial relationships that could be construed as a potential conflict of interest.
