# Supplementary material for: Evaluation of the Implementation of a 25-Year Outdoor School Ground Smoking Ban: A Qualitative Interview Study With Implications for Prevention Practise
Source: Front Public Health. 2021 Apr 1;9:628748. doi: 10.3389/fpubh.2021.628748 (PMC8046920; doi:10.3389/fpubh.2021.628748)
Supplement: Supplementary file 1 [file Data_Sheet_1.docx]

# Supplementary material

**Table 1.** Municipalities in Stockholm with size, number of various upper secondary schools and education level of inhabitants (Municipalities included in the study are italicized.).

| Municipality name | Inhabitants (N)a | Public schools (N)b | Independent schools (N)b | Upper secondary schools - total (N) b | Inhabitants aged 25-64 years with ≥3 years university education (%)a |
| --- | --- | --- | --- | --- | --- |
| *Municipality A* | 974 073 | 35 | 72 | 107 | 42 |
| *Municipality B* | 112 48 | 5 | 2 | 7 | 30 |
| *Municipality C* | 105 189 | 4 | 8 | 12 | 40 |
| *Municipality D* | 98 979 | 5 | 9 | 14 | 20 |
| *Municipality E* | 94 606 | 4 | 2 | 6 | 20 |
| *Municipality F* | 92 095 | 3 | 0 | 3 | 19 |
| *Municipality G* | 82 429 | 1 | 12 | 13 | 47 |
| *Municipality H* | 79 990 | 1 | 1 | 2 | 29 |
| *Municipality I* | 73 857 | 1 | 5 | 6 | 41 |
| *Municipality J* | 71 874 | 2 | 6 | 8 | 45 |
| *Municipality K* | 62 622 | 1 | 4 | 5 | 16 |
| *Municipality L* | 52 414 | 1 | 3 | 4 | 35 |
| Municipality M | 48 964 | 1 | 2 | 3 | 20 |
| *Municipality N* | 48 333 | 1 | 0 | 1 | 27 |
| Municipality O | 48 123 | 1 | 2 | 3 | 47 |
| *Municipality P* | 46 786 | 2 | 0 | 2 | 24 |
| Municipality Q | 45 574 | 1 | 1 | 2 | 27 |
| Municipality R | 45 000 | 1 | 2 | 3 | 27 |
| Municipality S | 34 090 | 1 | 1 | 2 | 29 |
| *Municipality T* | 32 857 | 1 | 3 | 4 | 58 |
| Municipality U | 29 346 | 1 | 0 | 1 | 22 |
| Municipality V | 28 690 | 1 | 0 | 1 | 32 |
| *Municipality W* | 28 575 | 1 | 0 | 1 | 16 |
| *Municipality X* | 16 750 | 1 | 0 | 1 | 28 |
| Municipality Y | 12 003 | 0 | 0 | 0 | 37 |
| Municipality Z | 11 014 | 0 | 1 | 1 | 22 |
| **Mean*** | **854 564** | **2.9** | **5.2** | **8.1** | **31** |
| **Total** | **2 218 659** | **76** | **136** | **212** | **Not rel.** |

*The municipalities are sorted by size

a) Source: <https://www.scb.se/hitta-statistik/statistik-efter-amne/befolkning/befolkningens-sammansattning/befolkningsstatistik/pong/tabell-och-diagram/helarsstatistik--kommun-lan-och-riket/folkmangd-i-riket-lan-och-kommuner-31-december-2019-och-befolkningsforandringar-2019/>

b) Source: www.gymnasieguiden.se

**Table 2.** Example of analysis.

| Meaning unit | Condensed meaning unit | Code | Sub-category | Main category | Key concept |
| --- | --- | --- | --- | --- | --- |
| It doesn’t work! I have been working at a lot of different upper secondary schools and it has never worked. […] Young people do dangerous things and it doesn’t help with a ban. Point. | The principal doubts whether a law is an effective way to prevent students from smoking. | Factors connected to school-leader/leadership inhibit the implementation of the ban | School leadership | Smoking-ban implementation factors | Barrier |

**Table 3.** Perceived facilitators, barriers and potential to implement an

outdoor upper secondary school ground smoking ban.

| Main categories | Sub-categories | Codes | Key concepts |
| --- | --- | --- | --- |
| Smoking-ban implementation factors | Regulation of outdoor school ground smoking ban by The Tobacco Act | Regulation of outdoor school ground smoking ban by the Tobacco Act facilitates the implementation process | Facilitator |
|  |  | Regulation of outdoor school ground smoking ban by the Tobacco Act is inefficient | Barrier |
|  |  | Regulation of outdoor school ground smoking ban by the Tobacco Act could/should be refined | Potential |
|  | Enforcement actors | Factors connected to enforcement actors facilitates the implementation process | Facilitator |
|  |  | Factors connected to enforcement actors inhibit the implementation process | Barrier |
|  |  | Factors connected to enforcement actors could/should be developed | Potential |
|  | Municipality-based control of compliance with outdoor school ground smoking ban | Municipality-based control of compliance with school ground smoking ban is carried out properly | Facilitator |
|  |  | Municipality-based control of compliance with school ground smoking ban is inefficient | Barrier |
|  |  | Municipality-based control of compliance with school ground smoking ban could/should be improved | Potential |
|  | School leadership | Factors connected to school-leader/leadership facilitates the implementation process | Facilitator |
|  |  | Factors connected to school-leader/leadership inhibit the implementation process | Barrier |
|  |  | Factors connected to school-leader/leadership could/should be strengthened | Potential |
|  | School-based implementation and enforcement | School-based implementation and enforcement of the school ground smoking ban is carried out properly | Facilitator |
|  |  | School-based implementation and enforcement of the school ground smoking ban is inefficient | Barrier |
|  |  | School-based implementation and enforcement of the school ground smoking ban could/should be improved | Potential |
| School factors | School culture | The school culture facilitates the implementation process | Facilitator |
|  |  | The school culture inhibits the implementation process | Barrier |
|  |  | The school culture could/should be improved | Potential |
|  | School ground | The school ground facilitates the implementation process | Facilitator |
|  |  | The school ground or the absence of a school ground inhibits the implementation process | Barrier |
|  |  | The design of the school ground could/should be improved | Potential |
| Community environment factors | Other actors or activities in the society | Other actors or activities in the society facilitate the implementation process | Facilitator |
|  |  | Other actors or activities in the society inhibit the implementation process | Barrier |
|  |  | Other actors or activities in the society could/should be engaged/developed | Potential |
|  | Social environment | The social environment facilitates the implementation process | Facilitator |
|  |  | The social environment inhibits the implementation process | Barrier |
|  |  | The social environment could/should be changed | Potential |
|  | Laws and regulations apart from the smoking ban on school grounds | Laws and regulations apart from the smoking ban on school grounds inhibit the implementation process | Barrier |
|  |  | Laws and regulations apart from the smoking ban on school grounds could/should be refined or developed | Potential |
